# Comparing analgesic effect of regional blocks after hip arthroscopy

**DOI:** 10.1093/jhps/hnae006

**Published:** 2024-02-05

**Authors:** Xue Gao, Fu-Shan Xue, Cheng-Wen Li

**Affiliations:** Department of Anesthesiology, Beijing Friendship Hospital, Capital Medical University, Beijing 100050, People’s Republic of China; Department of Anesthesiology, Beijing Friendship Hospital, Capital Medical University, Beijing 100050, People’s Republic of China; Department of Anesthesiology, Beijing Friendship Hospital, Capital Medical University, Beijing 100050, People’s Republic of China

## TO THE EDITOR

In a randomized controlled trial with 50 patients undergoing hip arthroscopy, Yuan *et al*. [1] compared postoperative analgesic efficacy of the ultrasound-guided quadratus lumborum block (QLB) and lumbar plexus block (LPB) and showed that the QLB provided similar and good postoperative analgesia, with less influence on motor function and fewer complications. Given that the use of a multimodal analgesia strategy including regional blocks can improve pain control with decreased opioid consumption and is being emphasized in current clinical practice of enhanced recovery after surgery protocols for hip surgery [2], this study has potential implications. However, we noted several issues in this study that need further clarifications.

First, the authors did not provide preoperative neuropsychiatric status, pain levels and analgesic use in demographic data of patients. Available evidence indicates that preoperative poor psychosocial health status, pain severity and analgesic use are significantly associated with the occurrence of acute pain after hip arthroscopy [3, 4]. Thus, we are concerned that any significant between-group unbalance in these unknown preoperative factors would have biased their main and secondary outcomes.

Second, after 30 min of two blocks, quadriceps muscle strength in the affected limb was measured to compare their motor preservative effects. As preserving motor function aims to promote early postoperative ambulation and effect of regional blocks on motor function can decrease with time, however, we argue that a more reasonable study design should be to compare the motor preservative effects of two blocks in early postoperative period, as performed in previous work assessing postoperative analgesic efficacy of regional blocks in patients undergoing lower limb surgery [5].

Third, other than regional blocks, patient-controlled intravenous analgesia (PCIA) with sufentanil was also used for postoperative pain control. The opioid consumption for PCIA within 48 h postoperatively was not significantly different between groups, but the readers were not provided the time to first pressing PCIA pump and number of effective pressing PCIA pump. In fact, these PCIA variables are very important for comparing performance of regional blocks with a single injection, as their duration is not expected to be longer than 24 h [6].

Finally, this study observed the occurrence of postoperative complications but not any important outcome variable of enhanced recovery after surgery protocols for hip surgery [2], such as patient satisfaction with postoperative analgesia, time to early mobilization, time to hospital discharge and quality of early postoperative recovery. Because of this design limitation, an important issue that this study cannot answer is whether the QLB compared with the LPB can improve early postoperative experience and outcomes of patients undergoing hip arthroscopy.
